# Axitinib in combination with radiotherapy for advanced hepatocellular carcinoma: a phase I clinical trial

**DOI:** 10.1186/s13014-020-01742-w

**Published:** 2021-01-20

**Authors:** Kai-Lin Yang, Mau-Shin Chi, Hui-Ling Ko, Yi-Ying Huang, Su-Chen Huang, Yu-Min Lin, Kwan-Hwa Chi

**Affiliations:** 1 Department of Radiation Therapy and Oncology, Shin Kong Wu Ho-Su Memorial Hospital, Shih-Lin District, No. 95, Wen-Chang Road, Taipei City, 111 Taiwan; 2 School of Medicine, Fu Jen Catholic University, No. 510, Chung-Cheng Road, Hsin-Chuang, New Taipei City, Taiwan; 3 Division of Gastroenterology, Department of Internal Medicine, Shin Kong Wu Ho-Su Memorial Hospital, Taipei City, Taiwan; 4 Department of Biomedical Imaging and Radiological Sciences, National Yang-Ming University, No. 155, Sec. 2, Linong Street, Beitou District, Taipei City, Taiwan

**Keywords:** Advanced hepatocellular carcinoma, Axitinib, Maximum tolerated dose, Radiotherapy

## Abstract

**Background:**

To investigate maximum tolerated dose (MTD) of axitinib, a selective vascular endothelial growth factor receptor 1–3 inhibitor, in combination with radiotherapy (RT) for advanced hepatocellular carcinoma (HCC).

**Methods:**

This phase I study followed the rule of traditional 3 + 3 design. Major eligibility included: (1) patients with advanced HCC unsuitable for surgery, radiofrequency ablation or transarterial chemoembolization, or who failed after prior local–regional treatment; (2) failure on sorafenib or no grant for sorafenib from health insurance system. Eligible patients with advanced HCC received axitinib for total 8 weeks during and after RT. Three cohorts with axitinib dose escalation were planned: 1 mg twice daily (level I), 2 mg twice daily (level II) and 3 mg twice daily (level III). The prescribed doses of RT ranged from 37.5 to 67.5 Gy in 15 fractions to liver tumor(s) and were determined based on an upper limit of mean liver dose of 18 Gy (intended isotoxic RT for normal liver). The primary endpoint was MTD of axitinib in combination with RT. The secondary endpoints included overall response rate (ORR), RT in-field response rate, acute and late toxicities, overall survival (OS) and progression free survival (PFS).

**Results:**

Total nine eligible patients received axitinib dose levels of 1 mg twice daily (n = 3), 2 mg twice daily (n = 3) and 3 mg twice daily (n = 3). Dose-limiting toxicity (DLT) did not occur in the 3 cohorts; the MTD was defined as 3 mg twice daily in this study. ORR was 66.7%, including 3 complete responses and 3 partial responses, at 3 months after treatment initiation. With a median follow-up of 16.6 months, median OS was not reached, 1-year OS was 66.7%, and median PFS was 7.4 months.

**Conclusions:**

Axitinib in combination with RT for advanced HCC was well tolerated with an axitinib MTD of 3 mg twice daily in this study. The outcome analysis should be interpreted with caution due to the small total cohort.

*Trial registration* ClinicalTrials.gov (Identifier: NCT02814461), Registered June 27, 2016—Retrospectively registered, https://clinicaltrials.gov/ct2/show/NCT02814461

## Background

The management of inoperable hepatocellular carcinoma (HCC) is challenging. Local ablation treatments including radiofrequency ablation (RFA) or other ablative approaches can typically achieve excellent local control for tumors less than 3 cm [1, 2]. For large or multifocal tumors, regional therapy with transarterial chemoembolization (TACE) are commonly recommended. In randomized studies, patients receiving TACE had better survival than those treated with only symptomatic treatment [3, 4]. However, either local ablation or TACE is sometimes contraindications for reasons, such as large tumor size, large number, inadequate location, macrovascular involvement, or impaired liver function [4–9].

Advances in radiotherapy (RT) technique have made RT become more important in the treatment of inoperable HCC [10–24]. For example, intensity-modulated radiotherapy (IMRT) has improved conformity of tumor dose and can spare critical normal organs better, while image-guided radiotherapy (IGRT) and breathing motion management allow accurate RT delivery by reducing setup error and effect of breathing cycle on liver location [25, 26]. These together also lead to an emerging role of stereotactic ablative body radiotherapy (SABR) in HCC [27]. Considering tumor size and normal tissue tolerance, radiation doses have ranged widely. Therefore, for advanced HCC treated with RT, the outcomes were reported in a wide range, including local control from 50 to 70% and median survival from 6 to 18 months. Higher radiation doses, hypofractionated RT or SABR may improve these treatment outcomes. However, first recurrence was usually identified at an intrahepatic site beyond irradiated field [28]. A treatment strategy combining RT with systemic therapy may be indicated.

Sorafenib, a multi-kinase inhibitor against angiogenesis and tumor proliferation, has become the standard systemic therapy for advanced HCC after two randomized controlled trials proved better survival of patients treated with sorafenib than placebo [29, 30]. Regorafenib, another multi-kinase inhibitor similar to sorafenib, was approved as a second-line treatment for HCC after failure from sorafenib [31]. Lenvatinib, a new multi-kinase inhibitor, was recently approved as another first-line treatment of HCC after a randomized phase III study proved non-inferiority in term of overall survival compared with sorafenib [32]. However, a substantial portion of patients treated with these kinase inhibitors encountered intrahepatic progression eventually. It has been believed that adding local treatment to effective systemic therapy may possibly consolidate at least local therapeutic effect. Sorafenib in combination with RT was considered effective in tumor response [33, 34], but potential hepatic toxicities may undermine the benefit of the strategy [35].

At the timepoint when our present study was initiated, sorafenib was the only approved targeted therapy for advanced HCC. Meanwhile, axitinib, a potent kinase inhibitor selectively inhibiting vascular endothelial growth factor (VEGF) receptors 1, 2 and 3, demonstrated superior outcomes for renal cell carcinoma (RCC) when compared with sorafenib, and thus axitinib was approved as second-line treatment for advanced RCC after failure of prior treatment with sunitinib or a cytokine [36]. HCC and RCC are both hypervascular cancers that can be potentially controlled by angiogenesis inhibitor. Axitinib was also studied for HCC in some clinical trials. In a randomized placebo-controlled phase II trial for locally advanced or metastatic HCC who failed from sorafenib, axitinib improved progression-free survival and showed overall response rate of 9.7%, but did not demonstrated benefit in overall survival [37]. Another phase II trial also reported second-line axitinib showed encouraging response rate with well tolerability [38].

Preclinical studies suggested axitinib can increase apoptosis of tumor endothelial cells after RT in vitro [39]. Some in vivo studies also demonstrated axitinib may effectively and safely improve tumor control with RT [39, 40]. Axitinib in combination with RT seems to be a potential approach. We hypothesized RT combined with axitinib would be safe and effective for advanced HCC, but the safety profile is not yet established. This phase I study aimed at determining the safety and maximum tolerated dose (MTD) of axitinib in combination with radiotherapy for advanced hepatocellular carcinoma.

## Methods

This phase I study was approved by the institutional review board (No. 20150704 M) and was registered in ClinicalTrials.gov (Identifier: NCT02814461). Patients with advanced HCC unsuitable for resection, liver transplantation, RFA or TACE, or who failed after prior local–regional treatment were eligible. Other key eligibility criteria included failure on sorafenib or no grant for sorafenib from health insurance system, Child–Pugh score A or B, and ECOG performance status 0–2. Multiple tumors, portal vein thrombosis, regional lymph node metastasis or distant metastasis was allowed. Major exclusion criteria included high risk of bleeding (e.g. active peptic ulcer, unstable esophageal/gastric varices, history of aneurysm, and requirement of anticoagulant therapy) and pre-existing uncontrolled hypertension (systolic > 140 mmHg, diastolic > 90 mmHg) or proteinuria ≥ 500 mg/24 h.

This phase I study followed the rule of traditional 3 + 3 design, and dose escalation of axitinib was conducted with 3 dose levels: 1 mg twice daily (level I), 2 mg twice daily (level II) and 3 mg twice daily (level III). Because the interaction between axitinib and RT was not well known before this study, the starting dose of axitinib was set at a minimal dose of 1 mg twice daily for the best of safety. The regimen of RT was 37.5 to 67.5 Gy in 15 fractions in 3 weeks (2.5 to 4.5 Gy per fraction) to liver tumor(s) (e.g. portal vein thrombosis, tumors with size ≥ 3 cm, or recurrent/refractory tumors). The final prescribed dose of RT was based on an upper limit of mean liver dose of 18 Gy for all plans (intended isotoxic RT for normal liver). Daily Entecavir 0.5-1 mg or Telbivudine 600 mg was recommended for patients with hepatitis B during and 3 months after RT. The primary endpoint was MTD of axitinib in combination with RT for advanced HCC. Secondary endpoints included overall response rate (ORR), RT in-field response rate, acute and late toxicities, overall survival (OS) and progression free survival (PFS). The RT in-field response rate was defined as the response rate of the irradiated tumor(s) within planning target volume of RT.

Dose-limiting toxicity (DLT) was defined as, according to the Common Terminology Criteria for Adverse Events (CTCAE) version 4.0, any of the following when considered related to protocol treatment: any grade 4 or 5 toxicities, grade 3 gastrointestinal toxicity despite the use of medical intervention and/or prophylaxis, grade 3 anemia, or grade 3 nonhematologic toxicities except nausea, vomiting, diarrhea, constipation, pain, and hypertension controlled with medication. In the beginning of the study, the first 3 patients were treated at starting dose of axitinib with 1 mg twice daily, and the next step would follow the rule described here. In order to observe any acute or delayed toxicities, our investigators waited for at least 3 months before moving to subsequent dose levels. If DLT was observed in 0 of 3 patients at a given dose level, the study would enter the next higher dose level. If DLT developed in ≥ 2 of 3 patients, the study would return to the next lower dose level if any. If DLT was noticed in 1 of 3 patients at a given dose level, additional 3 patients would be needed at this dose level. If DLT was noticed in 1 patient of the expanded 6-patient cohort, the study proceeded to the next higher level. If DLT developed in ≥ 2 patients of the expanded 6-patient cohort, the trial would proceed to the next lower dose level if any. When there were only 3 patients in the next lower dose level, 3 additional patients would be enrolled; while 6 patients are already there, the phase I trial would be stopped. MTD is defined as the dose at which ≤ 1/6 encounters DLT. It was estimated that about 9 to 18 patients would be enrolled in the phase I study. At least 3 months of follow-up after completion of protocol treatment should be performed to allow an adequate observation of DLT occurrence.

The descriptive statistics were summarized as percentages for proportions and as median (with ranges in parentheses) for continuous values. By response evaluation criteria in solid tumors (RECIST) version 1.1 [41], the response were evaluated by a radiologist at 3 months after treatment initiation. Survival curves were analyzed by Kaplan–Meier method, using Log-rank test when determining statistical significance of difference between subgroups. A *p* value < 0.05 (two-tailed) would be considered statistically significant. All statistical analyses were performed using Statistical Package for Social Sciences software version 20 (SPSS, Inc, Chicago, IL).

## Results

During the phase I study, total nine patients were enrolled. Among the total 9 patients, median age was 72 years (range 37–83 years), 88.9% were male, 78% had Child–Pugh class A, and 67% had hepatitis B or C (Table 1). At baseline, 33% had multiple liver tumors, 33% had portal vein thrombosis, none had lymph node metastasis, 11% had distant metastasis, and the median of liver tumor maximum diameter was 6.6 cm (Table 1). Each 3 patients subsequently entered cohorts of axitinib dose levels: 1 mg twice daily (n = 3), 2 mg twice daily (n = 3) and 3 mg twice daily (n = 3). Dose-limiting toxicity (DLT) did not occur in the 3 cohorts (Table 2), and the MTD was defined as 3 mg twice daily in this study. The most common adverse events (AEs) occurring in patients receiving axitinib (all grades) were hypertension, proteinuria, increased alanine transaminase (ALT), increased alkaline phosphatase (ALK-P), and increased bilirubin. The most common grade 3 AEs were hypertension, which could be managed by anti-hypertensive agents. Other grade 3 AEs included nausea, vomiting and diarrhea, which were all manageable. Among all patients, no grade 4 or 5 AEs occurred.

**Table 1:** Patient and tumor characteristics at baseline (n = 9)

Age, years, median (range)	72 (37–83)
Gender (male: female)	8:1
ECOG performance status	
0	1 (11%)
1	8 (89%)
Child–Pugh score	
5 (class A)	4 (45%)
6 (class A)	3 (33%)
7 (class B)	2 (22%)
Etiology of HCC	
Hepatitis B virus (HBV)	5 (56%)
Hepatitis C virus (HCV)	1 (11%)
Non-HBV, non-HCV	3 (33%)
BCLC staging	
B	1 (11%)
C	8 (89%)
Number of liver tumor(s)	
Single	6 (67%)
Multiple	3 (33%)
Portal vein thrombosis	
Present	3 (33%)
Absent	6 (67%)
Regional lymph node metastasis	
Present	0 (0%)
Absent	9 (100%)
Distant metastasis	
Present	1 (11%)
Absent	8 (89%)
Maximum diameter of liver tumor, cm, median (range)	6.6 (2.3–12.3)
RT doses, Gy, median (range)	45 (37.5–53)

**Table 2:** Toxicities

	Total (n = 9)		Level I cohort (n = 3)		Level II cohort (n = 3)		Level III cohort (n = 3)	
	Grade 1–3	Grade 3	Grade 1–3	Grade 3	Grade 1–3	Grade 3	Grade 1–3	Grade 3
Leucopenia	4	0	0	0	2	0	2	0
Anemia	1	0	1	0	0	0	0	0
Thrombocytopenia	4	0	0	0	2	0	2	0
Increased ALT	5	0	2	0	1	0	2	0
Increased alkaline-P	6	0	3	0	0	0	3	0
Increased total bilirubin	6	0	2	0	1	0	3	0
Increased creatinine	2	0	1	0	1	0	0	0
Hypothyroidism	3	0	1	0	0	0	2	0
Proteinuria	6	0	2	0	1	0	3	0
Skin rash	1	0	0	0	0	0	1	0
Hand numbness	1	0	1	0	0	0	0	0
Hypertension	9	5	3	1	3	2	3	2
Nausea/vomiting	1	1	1	1	0	0	0	0
Diarrhea	3	2	1	1	1	1	1	0
Constipation	2	0	1	0	1	0	0	0

**Fig. 1:**
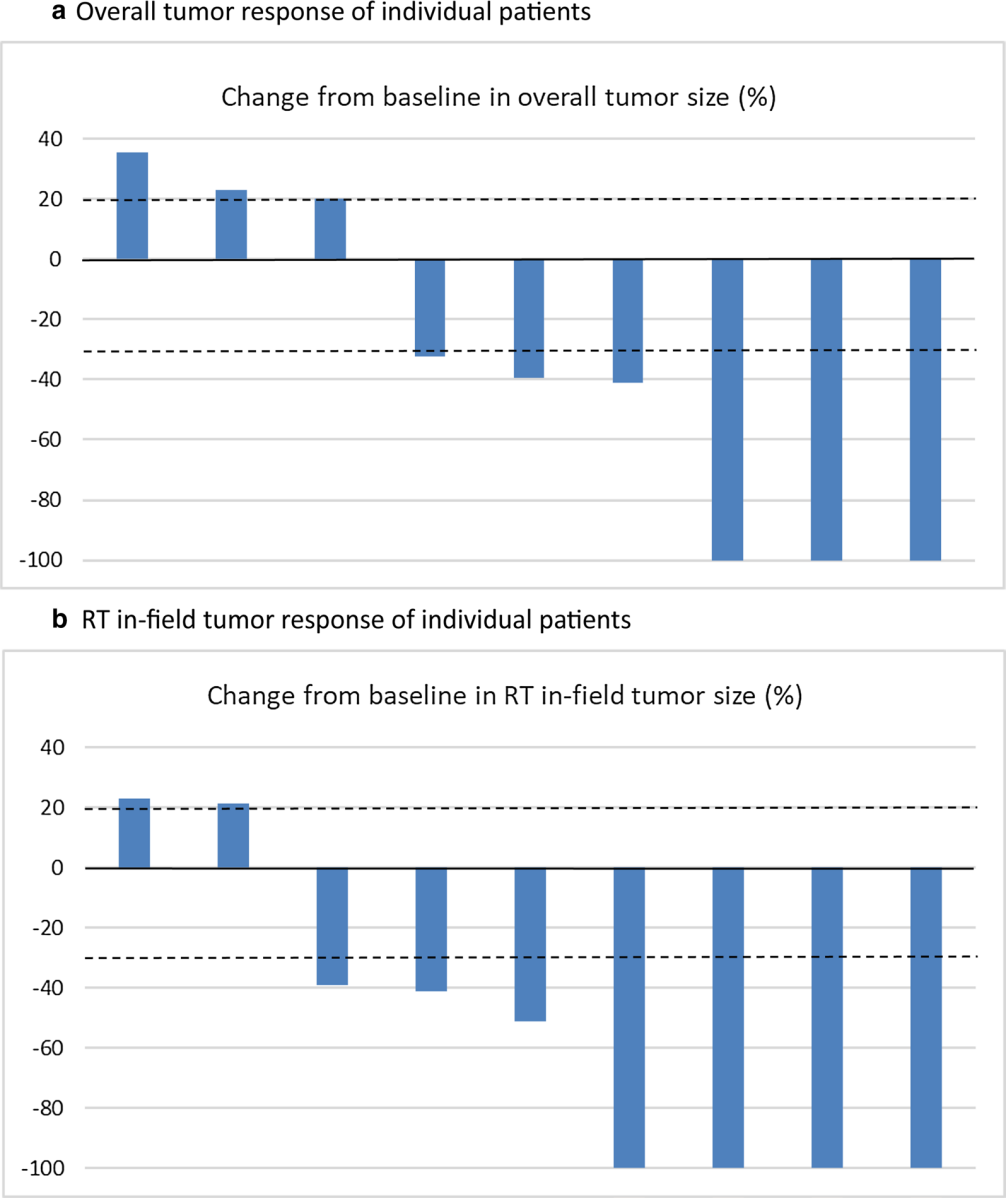
Waterfall plot for percentage change in tumor size at 3 months after treatment initiation. The dashed line at 20% means the cut-off value for progressive disease, and the dashed line at -30% for determination of means the cut-off value for partial response. **a** Overall tumor response of individual patients. **b** RT in-field tumor response of individual patients

**Fig. 2:**
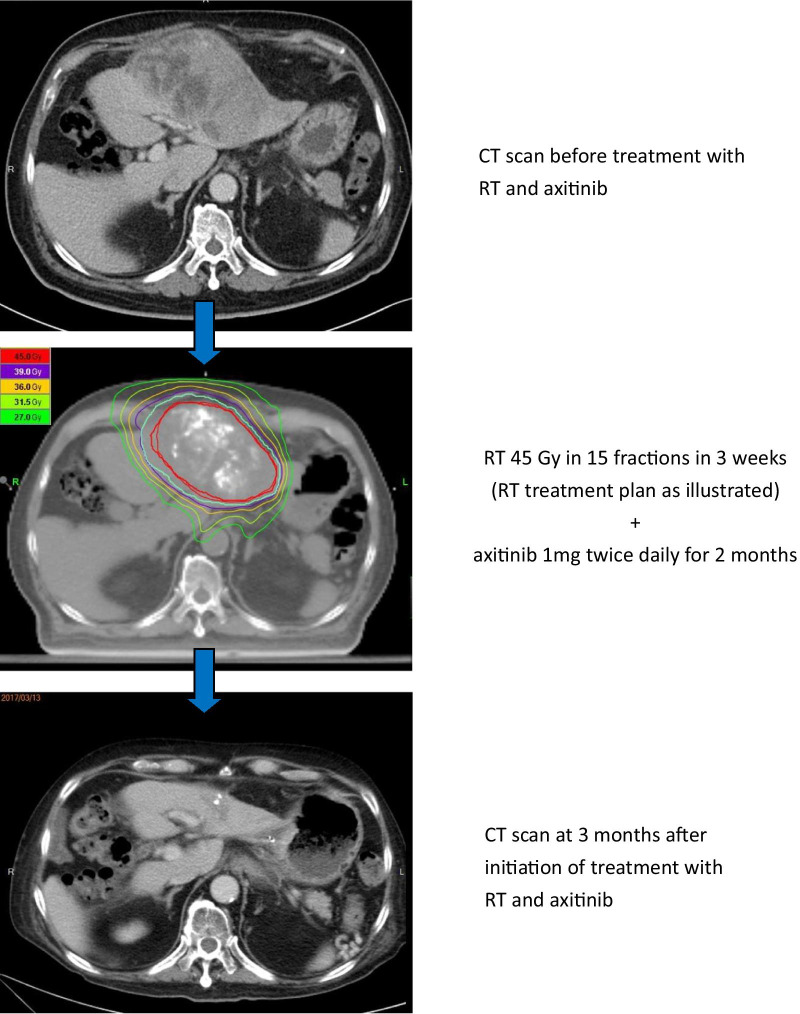
A case presentation (complete response, long-term alive, no recurrence). A 72-year-old man, with liver cirrhosis Child–Pugh class A (non-HBV, non-HCV related), was diagnosed as having a 12-cm HCC in left lobe of liver with classical enhancement pattern and involvement of left portal vein by CT scan in August 2016, clinically staged as cT3bN0M0, BCLC stage C. High alpha-fetoprotein (AFP) up to 160.1 ng/ml was noted. TACE failed with only partial obliteration of tumor vessels. He was then eligible for this phase I trial. He received RT with 45 Gy in 15 fractions plus axitinib 1 mg twice daily for 2 months. The patient tolerated the treatment well. At 3 months after RT initiation, follow-up CT scan revealed complete response of the tumor, and AFP decreased to 1.9 ng/ml. In 2020, the patient is still regularly followed up without recurrence

Among all 9 patients, overall response rate by RECIST criteria was 66.7%, including 3 complete responses (CR) and 3 partial responses (PR), at 3 months after treatment initiation (Table 3 & Fig. 1a). RT in-field response rate was 77.8% (4 CR and 3 PR) (Table 3 and Fig. 1b). The axitinib dose levels were not associated with tumor response (*p* = 0.406). Figure 2 illustrated one patient with CR with CT scans before and after RT in combination with axitinib 1 mg twice daily.

**Table 3:** Tumor response to axitinib in combination with radiotherapy (n = 9)

	Overall response	RT in-field response
Complete response (CR)	3 (33.3%)	4 (44.4%)
Partial response (PR)	3 (33.3%)	3 (33.3%)
Stable disease (SD)	0 (0%)	0 (0%)
Progressive disease (PD)	3 (33.3%)	2 (22.2%)
Response rate (CR + PR)	6 (66.7%)	7 (77.8%)

**Fig. 3:**
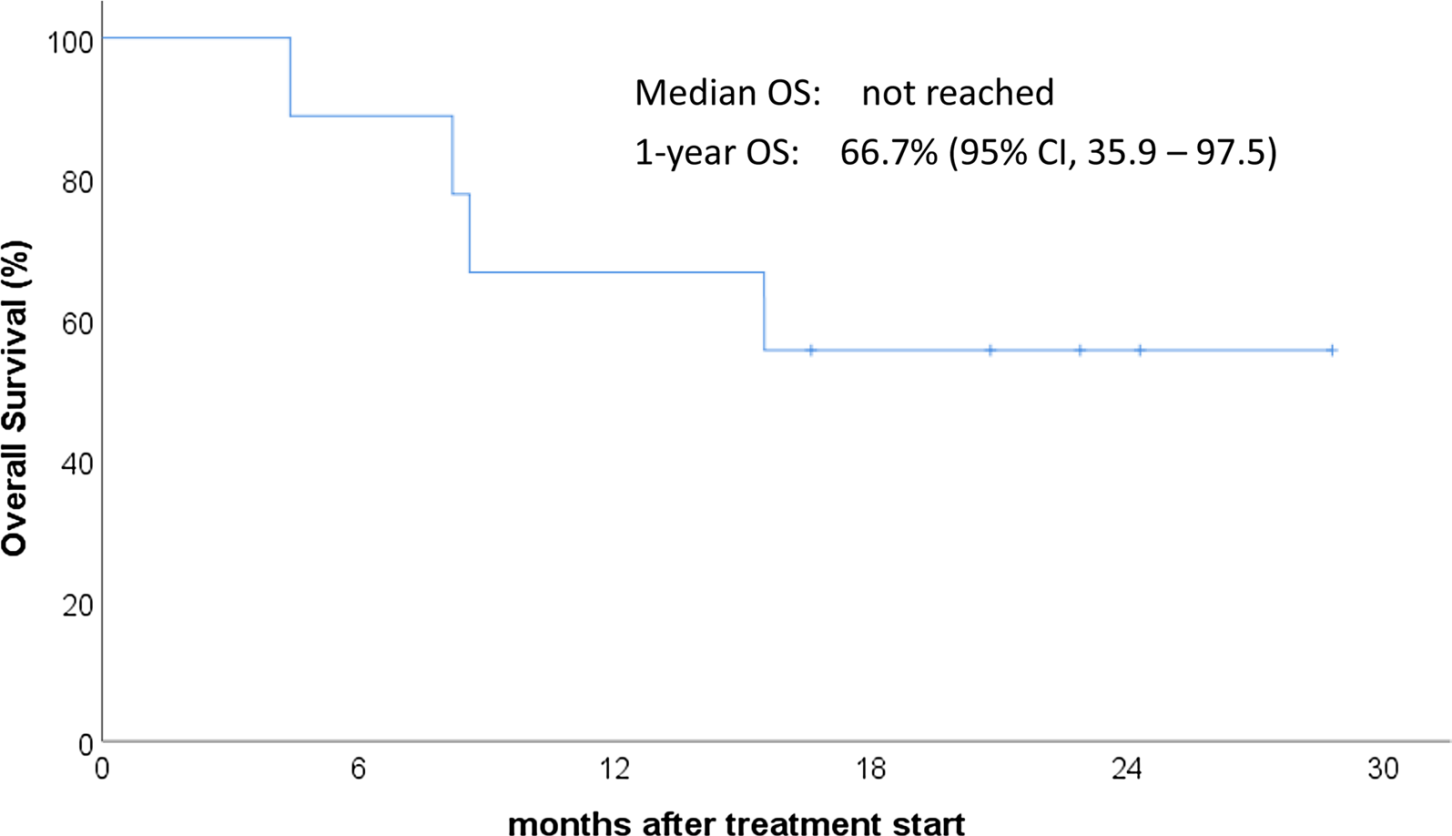
Overall survival

**Fig. 4:**
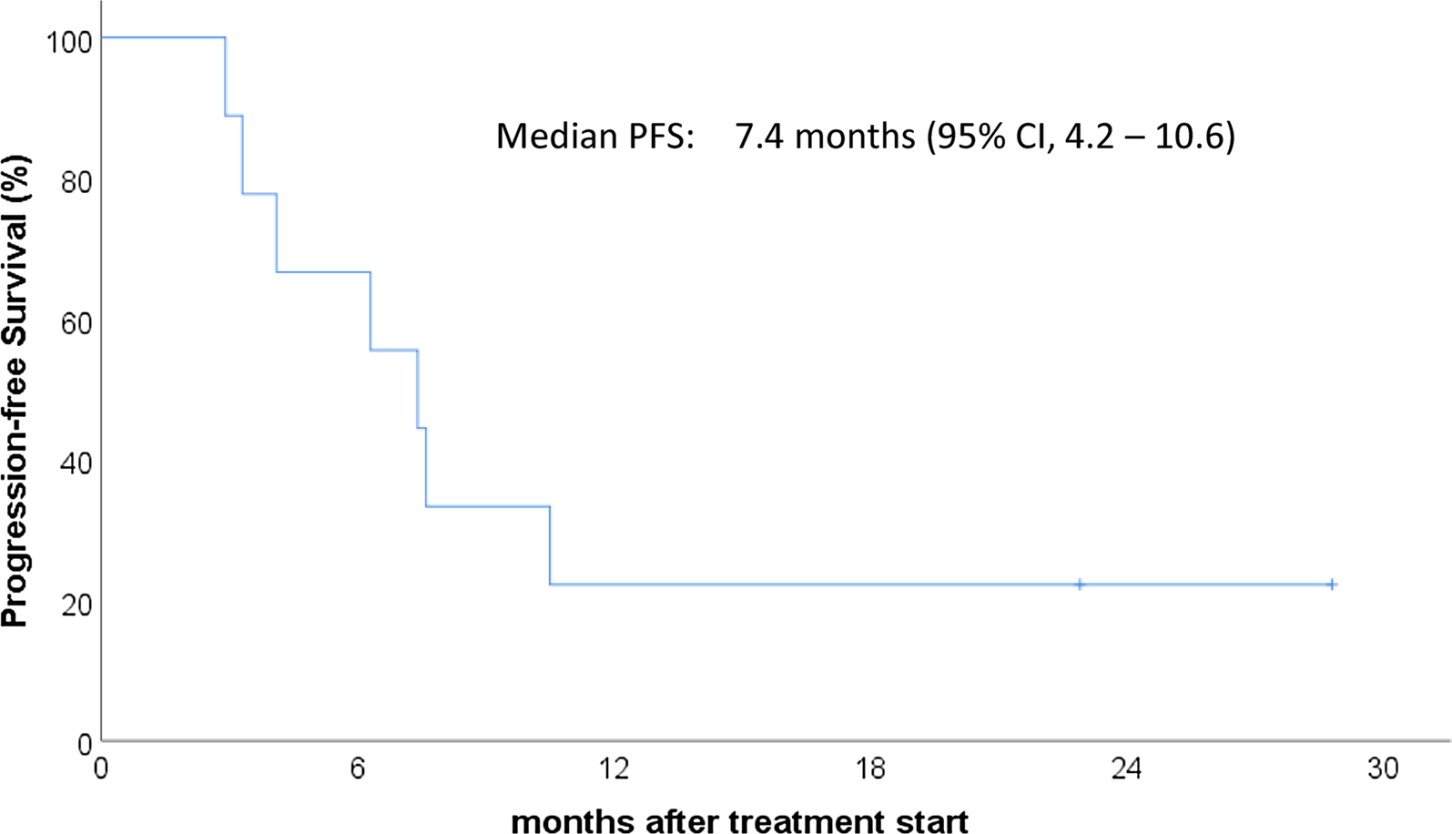
Progression-free survival

With a median follow-up of 16.6 months, median overall survival (OS) was not reached, 1-year OS was 66.7% (Fig. 3), and median progression-free survival (PFS) was 7.4 months (Fig. 4). On univariate analysis, responders (*p* = 0.024) and Child–Pugh A (*p* = 0.018) were associated with favorable OS. Responders (*p* = 0.002) and Child–Pugh A (*p* = 0.002) were also associated with favorable PFS.

## Discussion

Anti-angiogenic agents or transarterial therapies proved therapeutic value for HCC, a hypervascular cancer. Combination of some anti-angiogenic agents and RT showed potential benefit at both pre-clinical and clinical level [39, 40, 42–45]. In pre-clinical studies, anti-angiogenesis may allow better maturation of cancer blood vessels, and could potentially improve tumor oxygenation and thus tumoricidal effect of RT [46, 47]. According to in vitro and in vivo studies, axitinib can improve tumor control of RT by radiosensitizing tumor endothelial cells [39], and xenograft lung tumors on mice treated with axitinib and RT impressively showed complete tumor response and even reduced lung toxicity compared with RT or axitinib alone [40]. In our study, the response rate of axitinib in combination with RT is encouraging. This could be contributed by radiosensitization effect from addition of axitinib.

The safety of axitinib in combination with RT was not yet established before our study. Following a principle of the best safety, the starting dose of axitinib in our present phase I study was set as a minimal practicable dose of 1 mg twice daily, and the dose would be escalated by a relatively safe dose interval. In addition, we intended to deliver isotoxic and safe RT to normal liver with similar mean liver doses approaching 18 Gy for each patient. Although this caused heterogeneous prescribed RT dose in our study, this would be a necessary measure to make RT toxicities relatively constant and could enable appropriate evaluation of tolerability regarding axitinib MTD in combination with RT.

Our study successfully proved that axitinib in combination with RT is safe at least up to the dose of axitinib 3 mg twice daily, which was considered as the MTD in this study. The dose is already within the recommended dose range of axitinib: 2 mg twice daily to 10 mg twice daily adjusted according to individual tolerability [36, 48, 49]. According to our data, no additional toxicities were induced by the combination of RT and axitinib. All the AEs did not exceed grade 3 and were all manageable. We did not further escalate the dose because we had only limited resources for this study. If any other study groups want to conduct another similar phase I study, a starting dose with axitinib 3 mg twice daily can be considered. A determined MTD will facilitate design of a phase II study evaluating efficacy.

Clinical experiences with RT and anti-angiogenic agent showed some encouraging results. One retrospective study treated advanced HCC with RT and sunitinib reported objective response rate of 74% and a median survival of 16 months [50], which was compatible with the result of several phase I or II studies using sorafenib plus RT [35, 51]. RT in combination with effective systemic therapy may possibly exert the effect of spatial cooperation which may be translated to improved PFS and even OS. Our present study showed an acceptable PFS and impressive OS for advanced HCC treated with the combination strategy. However, due to only small cohort of patients, the efficacy reported in our phase I study should be interpreted with caution. Further phase II or even phase III study is required to adequately evaluate the efficacy. We are planning to conduct a phase II trial investigating the efficacy of this combination strategy.

Since regorafenib and lenvatinib were both proved as effective treatment for HCC [31, 32], the combination of RT with these relatively new agents could also be studied in the setting of clinical trial [52]. Adverse effects caused by regorafenib are serious concern because a substantial portion of HCC patients cannot well tolerate even regorafenib monotherapy [31]. Lenvatinib could be a better candidate to try a combination treatment with RT because many patients can better tolerate lenvatinib monotherapy as compared with sorafenib [32]. Several other new treatments for advanced HCC emerge recently, including ramucirumab or immunotherapy with immune checkpoint inhibitors. Various combination treatments are worthy of further research [52].

## Conclusions

Axitinib in combination with RT for advanced HCC is well tolerated with an axitinib MTD of 3 mg twice daily in this study. Some patients experienced tumor response to the protocol treatment, even with low dose of axitinib. However, the outcome analysis should be interpreted with caution due to the small total cohort.

## Data Availability

The datasets used and/or analyzed during the current study are available from the corresponding author on reasonable request.

## Abbreviations

HCC: Hepatocellular carcinoma
MTD: Maximum tolerated dose
RT: Radiotherapy
IMRT: Intensity-modulated radiotherapy
IGRT: Image-guided radiotherapy
SABR: Stereotactic ablative body radiotherapy
DLT: Dose-limiting toxicity
RFA: Radiofrequency ablation
TACE: Transarterial chemoembolization
VEGF: Vascular endothelial growth factor
RCC: Renal cell carcinoma
ORR: Overall response rate
OS: Overall survival
PFS: Progression free survival
RECIST: Response evaluation criteria in solid tumors
AFP: Alpha-fetoprotein
ALK-P: Alkaline phosphatase
CR: Complete responses
PR: Partial responses
